# Optimized Weight Low-Frequency Search Coil Magnetometer for Ground–Airborne Frequency Domain Electromagnetic Method

**DOI:** 10.3390/s23063337

**Published:** 2023-03-22

**Authors:** Fei Teng, Ye Tong, Bofeng Zou

**Affiliations:** College of Instrumentation & Electrical Engineering, Jilin University, Changchun 130012, China

**Keywords:** cupped flux concentrators, search coil magnetometers, optimized weight, low frequency, rugby ball winding

## Abstract

The vertical component magnetic field signal in the ground–airborne frequency domain electromagnetic (GAFDEM) method is detected by the air coil sensor, which is parallel to the ground. Unfortunately, the air coil sensor has low sensitivity in the low-frequency band, making it challenging to detect effective low-frequency signals and causing low accuracy and large error for interpreted deep apparent resistivity in actual detection. This work develops an optimized weight magnetic core coil sensor for GAFDEM. The cupped flux concentrator is used in the sensor to reduce the weight of the sensor while maintaining the magnetic gathering capacity of the core coil. The winding of the core coil is optimized to resemble the shape of a rugby ball, taking full advantage of the magnetic gathering capacity at the core center. Laboratory and field experiment results show that the developed optimized weight magnetic core coil sensor for the GAFDEM method is highly sensitive in the low-frequency band. Therefore, the detection results at depth are more accurate compared with those obtained using existing air coil sensors.

## 1. Introduction

With the development of industries, the demand for oil gas, iron, copper, and other important mineral resources became critical [1]. Mineral resources became an important basis for social development [2]. The ground–airborne frequency domain electromagnetic (GAFDEM) method is suitable for detecting resource distribution and geological structure by a grounded electrical source and an airborne receiver [3]. This technique is practical and suitable for large surveying areas with challenging ground conditions that are difficult or dangerous to access for traditional ground surveys [4]. For the measurement of the GAFDEM signal, primary electromagnetic fields may be generated by passing an alternating current through a long electrical wire or a large loop of wire [5]. The response of the conductor is to generate secondary electromagnetic fields, and the resultant fields may be detected in the airborne receiver coil by electromagnetic induction. The difference between the transmitted and received electromagnetic fields reveals the presence of the conductor and provides information on its geometry and electrical properties [6].

In the GAFDEM method, electromagnetic fields are attenuated during their passage through the ground, and their amplitude decreases exponentially with depth [7]. The depth of penetration increases when the frequency of the electromagnetic field and the conductivity of the ground decrease [8]. The frequency used in the GAFDEM survey can be tuned to a desired depth range in any particular medium [9,10]. However, the frequency dependence of the depth of penetration places constraints on the GAFDEM method [11]. In practical application, the low-frequency part of the signal is indispensable to complete deep exploration.

For GAFDEM instruments, most of the receiving sensors are air-core coils [12,13,14]. Airborne electromagnetic sensors, such as VTEM, ZTEM, and SKYTEM are all air coil sensors, and GAFDEM (semi-airborne) is a continuation of the airborne electromagnetic design [15,16,17]. The low-frequency response of the air-core coil is insufficient, resulting in limited detection depth. Owing to the load constraint of rotorcraft, the weight constraint should also be considered in the design of sensor. Therefore, the characteristics of the sensor influence the accuracy of the inversion results.

In this work, we develop an optimized weight search coil magnetometer based on a cupped flux concentrator with high sensitivity, low noise, and low weight. To improve the apparent permeability of the sensor, we add a cupped flux concentrator at both ends of the sensor. Compared with that of the same size flux concentrator, the mass is 25% lighter, which meets the load constraint of the aircraft. Simulation results show that the effect of the two flux concentrators is almost the same. Furthermore, we observe the non-uniform distribution of magnetic inductance lines on the magnetic core. Hence, we propose a rugby ball winding method to reduce the weight of sensor. To further verify the performance of the sensor, we perform a comparative experiment between the air-core coil and search coil magnetometers in the field. Compared with traditional sensors, our new sensor can effectively receive the low-frequency part of the GAFDEM signal and achieve the accurate inversion of the apparent resistivity and depth.

## 2. Materials and Methods

### Basic Principles of the Search Coil Magnetometer

The GAFDEM method is a ground-based arrangement of electrical emission source (direction x). A UAV is used in the air to carry a search coil to acquire the vertical component magnetic field. The GAFDEM method is similar to the ground-based controlled source electromagnetic method CSAMT. The emitted current (I) generates an excited magnetic field, causing the underground anomalies to generate eddy currents, and the secondary magnetic field is radiated into the air by the eddy currents.

The search coil and receiving system are lifted by the UAV. The receiving system records the secondary field response in the direction (y) perpendicular to the electrical emission source after data processing, thus enabling the detection of resistivity (ρ) at different depths underground. The coil is placed in the air at a height of |z|(z<0), and the magnetic field $B_{z}$ at R can be calculated as the z-component:

(1) $$B_{z}=\frac{\mu_{0}IL}{4\pi }\frac{y}{R}\int_{0}^{\infty }\left(1+r_{TE}\right)e^{u_{0}z}\frac{\lambda^{2}}{\mu_{0}}J_{1}\left(\lambda R\right)d\lambda$$

where $\mu_{0}=4\pi \times 10^{-7}H/m$, $\mu_{0}={\left(\lambda^{2}-{k_{0}}^{2}\right)}^{\frac{1}{2}}$, $R=\sqrt{\left[x^{2}+y^{2}\right]}$, and $r_{TE}$ is the reflection coefficient related to the resistivity and thickness, $k_{0}$ is the wavenumber in the air, λ is the integral variable, and $J_{1}$ is a first-order Bessel function of the first class. The detection of resistivity structures at different depths in an underground space can be achieved by measuring the magnetic field signals at specific locations in the air at different frequencies. The equation of skinning depth is as follows:

(2) $$\delta =\sqrt{\frac{2}{\mu \omega \sigma }}$$

where ω is the operating frequency,μ is the magnetic permeability of the ground, and σ is the conductivity of the ground. As *ω* goes lower, *σ* means greater. In other words, the lower the frequency of the electromagnetic signal, the greater the detection depth. However, hollow coils do not respond well at low frequencies. Low-frequency electromagnetic waves are transmitted deep into the earth, corresponding to information about the deep parts of the earth when σ is constant.

**Figure 1:**
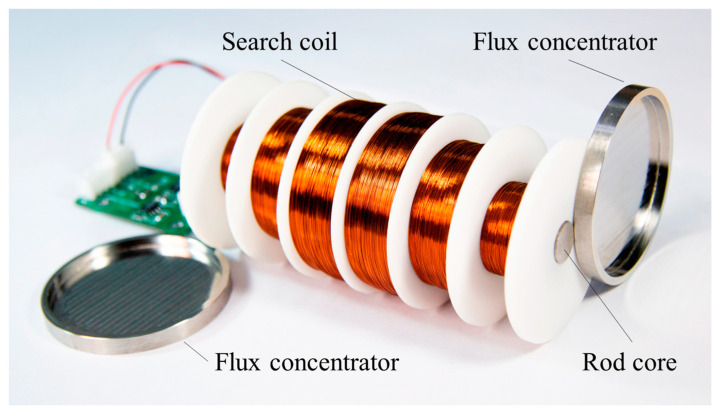
Experimental model of the search coil magnetometer.

The transmitter needs to emit electromagnetic fields of different frequencies and the sensors are placed in the measurement area to meet the conditions for receiving the magnetic field generated by the material in the ground. The sensor is considered to be the search coil magnetometer and is made up of basic units, including search coil and pre-amplifier. The flux concentrator and rod core are used to increase the gathering power of the magnetic field, thus enhancing the performance of the sensor. Its structure is shown in Figure 1. The search coil follows Faraday’s law of electromagnetic induction, and its induced electric potential is calculated as follows:

(3) $$e\left(t\right)=-n\frac{d\Phi \left(t\right)}{dt}=-\mu_{app}nGS\frac{dB\left(t\right)}{dt}.$$

**Figure 2:**
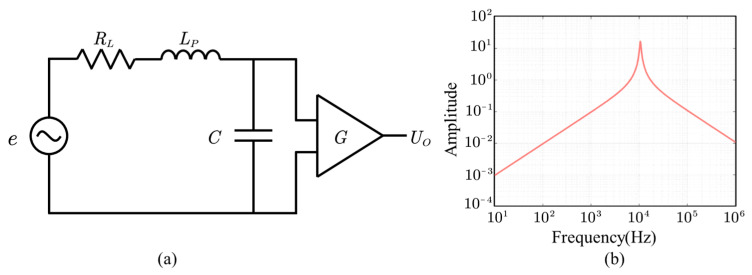
Equivalent circuit of the search coil (**a**) and its frequency response (**b**).

The physical model of the coil is shown in Figure 2a. Let n be the number of turns in the primary winding, and Φ the induction flux inside coils. The ratio between the magnetic field inside the core ($B_{in}$) and the external magnetic field outside the sensor ($B_{out}$) is called apparent permeability ($\mu_{app}$),$\;\mathrm{and}\;\mu_{app}nGS.$ is considered as the equivalent area of the search coil.

In the frequency domain, the electric potential of the coil is calculated as

(4) $$e=\mu_{app}\omega nGSB_{out}.$$

The frequency response of the coil is shown in Figure 2b and calculated as

(5) $$U_{O}=\frac{e}{1-\omega^{2}CL_{p}+j\omega R_{L}C}=\frac{j\omega NSG\mu_{app}B_{out}}{1-\omega^{2}CL_{p}+j\omega R_{L}C}$$

where $R_{L}$ denotes the resistance of coil, C denotes the capacitance of coil, and $L_{P}$ denotes the inductance of coil.

When air is inside the coil, the inductance of the coil is described by the following equation:

(6) $$L_{p}=\frac{\mu_{0}\sqrt{S/\pi }N^{2}}{2}\left[ln\left(\frac{4D\sqrt{S}}{\sqrt{lh}}\right)-1.75\right].$$

In summary, the magnetic fields of different frequencies can be picked up by the coil, and the strength of the received signal at different frequencies is related to the characteristics of the coil. The coil has a high-frequency sensitivity near the resonant frequency, and the performance of the coil can be maximized when the transmitting frequency is at the resonant frequency.

The reception frequency of the coil for the FEM is usually within 10 kHz. The sensitivity of the coil is maximum at the resonant frequency and gradually decreases when the frequency is far from the resonant frequency. Hence, the bandwidth of the coil is limited, especially at low frequency, where the signal detection capability is insufficient.

**Figure 3:**
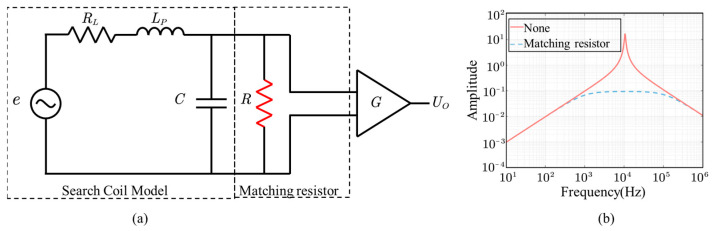
Equivalent circuit of the search coil with matching resistor (**a**) and its frequency response (**b**).

Therefore, we improve the model of the coil by connecting a resistor in parallel at the output as shown in Figure 3a. In this way, the risk of resonance is reduced and the bandwidth is increased. The amplitude–frequency characteristics of the improved coil are shown in Figure 3b. The response at low frequencies is still inadequate due to the coil resonance frequency at 10 kHz. As the coil has only one resonant frequency, the ground–space solenoid is usually sacrificed for the low-frequency response (less than 1 kHz) and it follows from this literature that even with matching resistors, it only changes the sensitivity of the high-frequency part [18].

## 3. Design and Results

### 3.1. Core Coil of Cupped Flux Concentration and the Shape of a Rugby Ball Winding

Adding a magnetic core to the center of the sensor effectively solves the problem of the low sensitivity of the coil at low frequencies. The internal core consisting of rod core and flux concentrator can amplify the external magnetic field, and the value of $\mu_{app}$ changes to greater than 1 [19]; $\mu_{app}$ depends only on the relative permeability of the magnetic material ($\mu_{r}$) and demagnetizing coefficient ($N_{d}$), where $N_{d}$ depends on the ratio of length to diameter (*m* = l/*d*).

(7) $$\mu_{app}=\frac{B_{in}}{B_{out}}=\frac{\mu_{r}}{1+\left(\mu_{r}-1\right)N_{d}}$$

(8) $$N_{d}=\frac{1}{m^{2}-1}\left[\frac{m}{\sqrt{m^{2}-1}}ln\left(m+\sqrt{m^{2}-1}\right)-1\right]$$

N coils are wound uniformly around a rod core whose length is 1, and $\mu_{app}B_{out}$ is considered the amplified magnetic field. Therefore, the electric potential of the coil is redefined as

(9) $$e=NS\left(\frac{\int_{0}^{l_{w}}\mu_{app}\left(l\right)dl}{L_{N}}\right)B_{out}\omega =NS{\overset{-}{\mu }}_{app}B_{out}\omega .$$

The above equation shows two ways to enhance a: one is to increase the effective receiving area (NS), and the other is to increase the apparent permeability ($\mu_{app}$).

When the effective area is certain, $\mu_{app}$ must be increased to raise the induced voltage of the coil.

(10) $$\mu_{app}=\frac{\mu_{r}}{1+\left(\mu_{r}-1\right)N_{d}\left(\frac{l_{w}}{D}\right)\frac{d^{2}}{D^{2}}}$$

This new structure reveals that the magnetic flux density of the rod core with flux concentrator becomes unevenly distributed compared with that of magnetic rods alone. The rod core with flux concentrator has the effect of collecting the magnetic field. We want to further lighten this effect by digging out a part of the structure in the center of the original flux concentrator.

**Figure 4:**
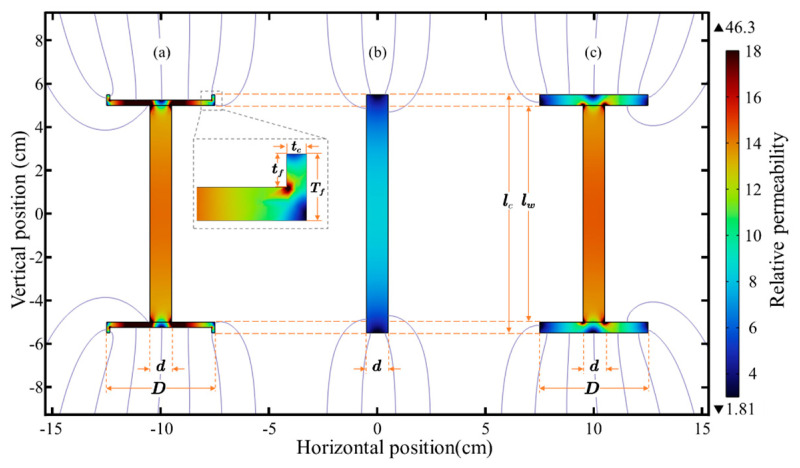
$\mu_{app}$ in different flux concentrators: (**a**) magnetic core with rod core and cupped flux concentrator, (**b**) magnetic core only with rod core, and (**c**) magnetic core with rod core and flux concentrator.

The new structure is shown in Figure 4.

(11) $$L_{P}=\frac{\mu_{0}\mu_{app}N^{2}S}{l_{c}}\left(2.11-1.1\frac{l_{w}}{l_{c}}\right)$$

**Figure 5:**
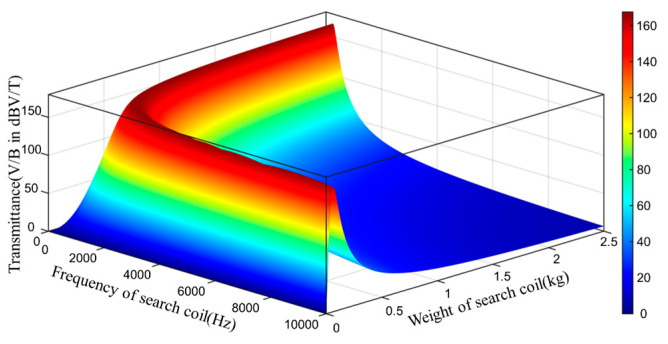
Transmittance of GAFDEM as a function of the weight of the search coil and the frequency of the search coil.

The inductance ($L_{P}$) of the coil is increased due to the addition of rod core and flux concentrator. Tightly wound coils increase capacitance. According to the resonant frequency equation, the resonant frequency of the coil is reduced. In addition, the resonant frequency is related to the weight of the coil, assuming that the average weight per turn is $\overset{-}{m_{N}}$ and the total mass is m. The resistance of the coil is

(12) $$R_{L}=\rho_{R}\frac{m}{\rho s^{2}}$$

where $\rho_{R}$ is the resistivity of the coil, ρ is the density of the wire, and s is the cross-sectional area of the wire. The above equation is brought into the sensitivity formula to obtain the relationship among sensitivity, weight, and resonant frequency. As the Figure 5. The resonant frequency exponentially increases when the mass increases linearly. The sensitivity is low at above 2 kHz, and the sensitivity only gradually becomes high at below 2 kHz.

**Figure 6:**
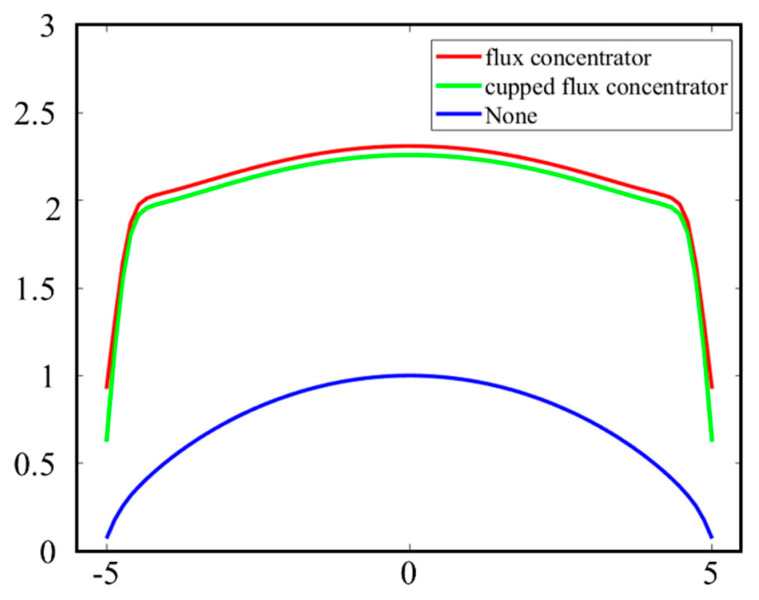
Comparison of magnetic field collection capacity of three core structures.

Unfortunately, it is difficult to describe the magnetic core with cupped flux concentrator by *Eq*. Thus, we perform simulations with finite element software to obtain the apparent permeability of the flux concentrator with different structures. As shown in Figure 6. The simulation results prove that the flux concentrator and cupped flux concentrator are almost identical. The cupped flux concentrator is reduced by 72.4 g, which is compared with the flux concentrator in terms of weight [20]. This new geometry reveals that the flux density along the rod core becomes more uniform compared with that along the magnetic core with only the rod core. However, $\mu_{app}$ is still the largest in the center and decreases in both sides.

On the basis of the structure described above and the distribution law of $\mu_{app}$, the winding method of the coil is optimized. The distribution of the rod core and that of $\mu_{app}$ on the bar is not uniform. If the coil is wound uniformly on the bar, then it migrates toward the center position and induces the maximum voltage. Meanwhile, the coils on both sides induce the minimum voltage. Therefore, the magnetic core is not fully exploited.

**Figure 7:**
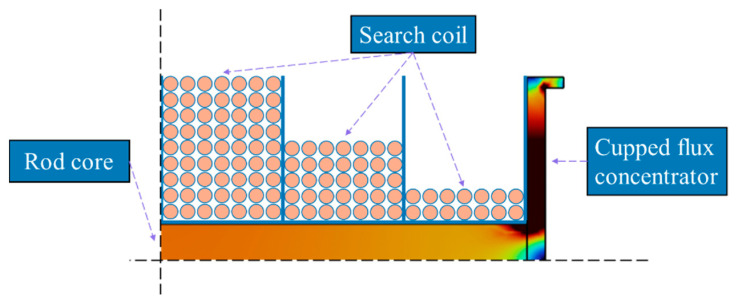
2D axisymmetric GAFDEM of the winding and magnetic core.

We want to take full advantage of the $\mu_{app}$ at the center to optimize the winding of the coil to resemble the shape of a rugby ball, with many turns being wound at the center of the rod core and less at the sides of the rod core. The 2D axisymmetric GAFDEM of the winding and magnetic core in Figure 7.

(13) $$e=\sum_{i=1}^{6}N_{i}S_{i}\left(\frac{\int_{L_{i}}^{L_{i+1}}\mu_{app}\left(l\right)dl}{\frac{L_{w}}{6}}\right)B_{out}\omega$$

### 3.2. Experimental Results

The experiment consists of two parts: laboratory and field experiments. For the laboratory experiment, the sensitivities of core coil and traditional air coil are compared inside an electromagnetically shielding laboratory. For the field experiment, we obtain the signals received by different coils and calculate different detection results according to the signals.

#### 3.2.1. Laboratory Experiment

After completing the design of two options for increasing sensitivity, we conduct a sensitivity test and compare it with the air coil. The results are shown in Table 1. An AC current source is used to provide excitation for the Helmholtz coils. This source produces a magnetic field of unchanging amplitude and adjustable frequency. The induced voltage of search coil magnetometers is detected by a dynamic signal analyzer.

**Figure 8:**
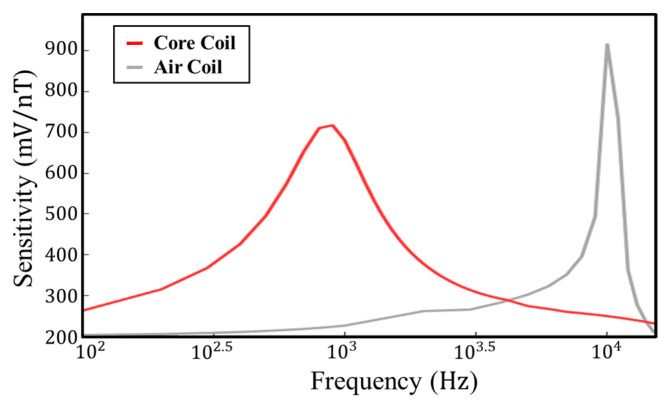
Sensitivity of air coil and core coil.

**Figure 9:**
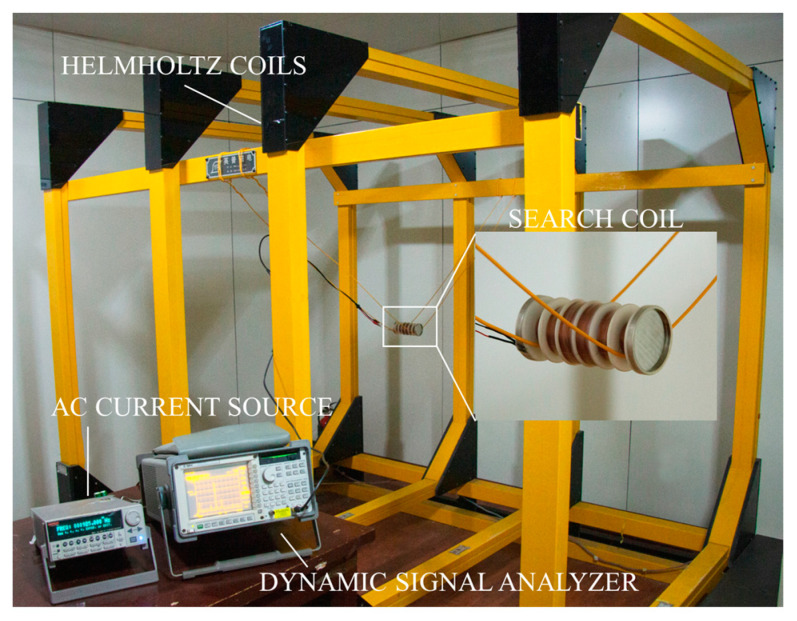
Overview of sensitivity experiment of core coil.

As shown in Figure 8, the response at 1 KHz is much higher for core coil than for air coil, and this phenomenon covers the range from 100 Hz to 6.5 KHz. The sensitivity of the core coil of this design is higher than that of the air coil at low frequencies. This search coil magnetometers makes up for the lack of response of the air coil sensor at low frequencies. At the same time, the weight is reduced by 144.8 g. The sensitivity experiment for the core coil is shown in Figure 9.

#### 3.2.2. Field Experiment

**Figure 10:**
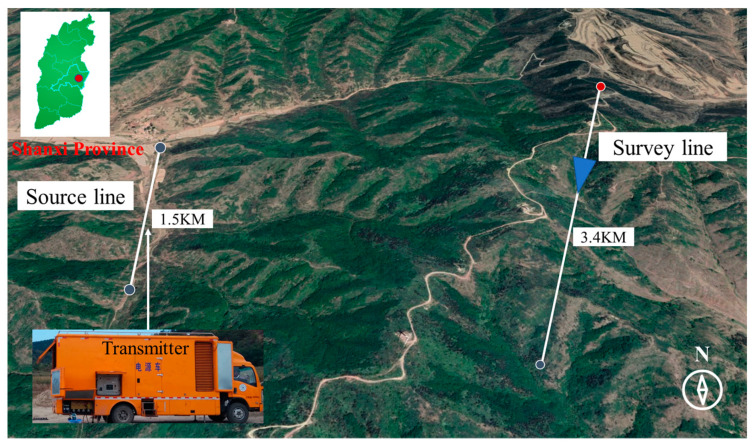
Location of the field experiment.

A series of field comparison studies is carried out to further confirm the efficacy of the suggested technique. The exploration site is located in Fuxin Coal, which is 1.5 km to the west of Pingding County. The mine is connected to the main highway of the county by a township road, 1.0 km from the Yangquan-Pingding County Secondary Highway and 8.0 km from Yangquan City, Shanxi Province as shown in Figure 10.

**Figure 11:**
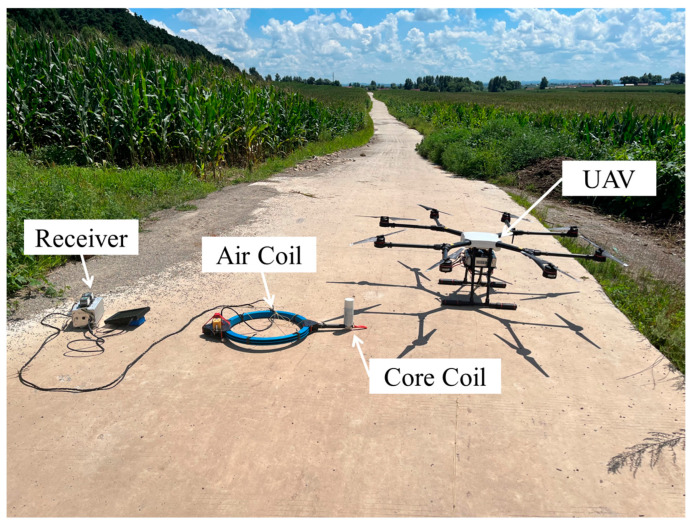
Overview of the field experiment.

**Figure 12:**
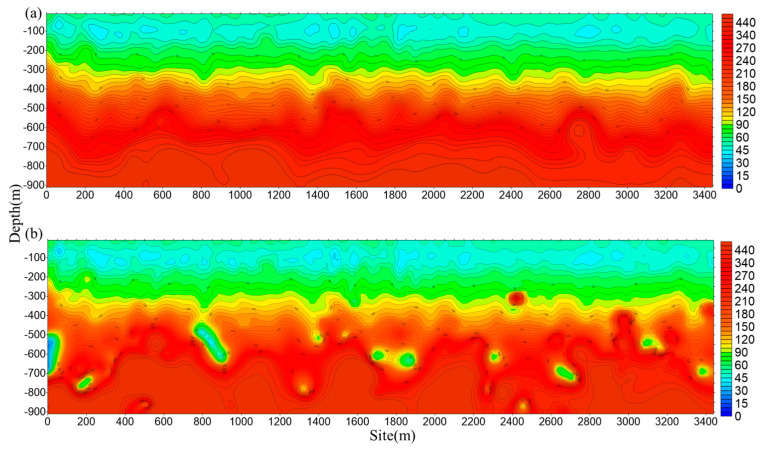
Comparison of detection performance utilizing different systems: (**a**) air coil and (**b**) air coil and core coil.

To verify the effectiveness of the core coil at low frequencies, we measure the core coil and the air coil as shown in Figure 11. The distance between the core coil (search coil magnetometer) and the axis of the air coil is set to 0.5 m to reduce the disturbance between them. The signals from the two different coils are received by the dual-channel receiver, and the transmitting system transmits in the frequency range of 10 Hz–10 kHz with a transmitting current of 30 A at the location shown in Figure 10. The electrical structure in the depth range of 10–900 m with different sensor solutions is shown in Figure 12. The same UAV flying the same route and flying at a speed of 5 m/s is used with the same receiver and transmitter system. A deficiency in the low-frequency response of the air coil is observed as shown in Figure 12a, particularly poor resolution at depths of 600 m to 900 m. Figure 12b shows the data from the air coil fused with the core coil. The resolution at depth is significantly higher than that captured by the air coil alone.

#### 3.2.3. Discussion

The center frequency of the air coil is higher than 2 kHz, so the detection capability of low-frequency electromagnetic signals is limited and directly affects the interpretation of deep geological information. Therefore, the signal-to-noise ratio of signals below 2 kHz needs to be improved. This work introduces a core coil capable of improving the low signal-to-noise ratio of low-frequency signals. The field experiment shows that the reception capability of the core coil at low frequencies is stronger than that of the air coil, giving the former a great advantage in the interpretation of deep geology.

However, the core coil and the air coil are fixed together and the energy of the core coil moving in the air is absorbed by the air coil for a large part. If the UAV alone carries the core coil in the air for measurement, then the interference from the motion is extremely serious. Therefore, further research should be conducted to reduce the noise interference caused by motion noise.

## 4. Conclusions

This study presents a lightweight, highly sensitive low-frequency search coil magnetometer. The core is designed in a cupped shape to reduce weight, and the wires are wound into a rugby ball shape for optimized weight and sensitivity. The search coil magnetometer has higher sensitivity at low frequencies than existing air coils. The low-frequency response of the core coil is verified in the laboratory, and field experiments prove its advantages in detecting great depths. Furthermore, the search coil magnetometer with core is used in the GAFDEM method for the first time.

## Figures and Tables

**Table 1:** Maximum detection time length according to the instrument detection threshold.

Type	Weight	Size	Sensitivity
Core coil	1.7 kg	∅5 cm	724 mV/nT @ 1 KHz
Air coil	2.4 kg	∅50 cm	227 mV/nT @ 1 KHz

## Data Availability

No new data were created or analyzed in this study. Data sharing is not applicable to this article.
